# A graph neural network model for inferring interindividual variation from experimental biological data

**DOI:** 10.1038/s41598-025-23320-4

**Published:** 2025-11-12

**Authors:** Fuminori Kawano

**Affiliations:** Graduate School of Health Science, Matsumoto University, 2095-1 Niimura, Matsumoto City, Nagano 390-1295 Japan

**Keywords:** Interindividual variation, Artificial intelligence, Machine learning, Deep learning, Computational biology and bioinformatics, Mathematics and computing, Neuroscience, Systems biology

## Abstract

Interindividual variation in biological responses to physiological stimuli is a widely recognized phenomenon. However, effective computational tools for identifying the individual-specific mechanisms remain limited. We present the bioreaction–variation network, a graph neural network (GNN) model designed to infer hidden molecular and physiological relationships underlying such variation in experimental biological data. To ensure applicability at a laboratory scale, the model was trained on a domain-specific corpus constructed from approximately 65 K published studies containing the keyword “skeletal muscle”. The architecture comprises five layers with a multi-head attention mechanism and a multi-layer perceptron, enabling the model to capture both local topological features and directional dominance between connected nodes. The GNN was trained to learn relationships from experimental models to target features, as well as among target features. Using real experimental input consisting of differential gene expression data from mouse skeletal muscle subjected to acute exercise, the model successfully inferred individualized networks, identifying both common and unique paths across individuals based on input experimental context. These results demonstrate the model’s capacity to extract interpretable, individual-specific biological connectivity patterns. The proposed framework serves as a proof of concept for customizable, context-based GNN inference designed to address biological variation at the individual level.

**Supplementary Information:**

The online version contains supplementary material available at 10.1038/s41598-025-23320-4.

## Introduction

Deep understanding of interindividual variation, including sex differences, in responses to physiological stimuli such as exercise, nutrition, aging, and pathological conditions is a critical step toward promoting lifelong health and enhancing the quality of human life. The Dunedin Multidisciplinary Health and Development Study, a longitudinal birth cohort study of individuals born in 1972–1973, has revealed substantial variability in the pace of aging among participants^1,2^. It further suggests that early-life psychological and nutritional exposures may induce long-term alterations in epigenetic mechanisms that contribute to interindividual differences in aging trajectories. Despite these findings, the processes by which such interindividual variation emerge and develop across the lifespan remain poorly understood. While regular physical activity is widely recognized for its beneficial effects on human health, it is also well documented that considerable variability exists in the outcomes of exercise interventions. For instance, Bamman et al.^3^ demonstrated that individuals exhibit heterogeneous responses to resistance training and can be categorized as extreme-, modest-, or non-responders, with these variations not attributable to sex or age. Similar variability has been reported in response to endurance training. Ross et al.^4^ reviewed data demonstrating that maximal oxygen consumption increased by only 7% in the lowest responder, whereas the highest responder exhibited an increase of 118% following 24 weeks of treadmill-based endurance training. Furthermore, Bonafiglia et al.^5^ quantitatively identified meaningful interindividual differences in trainability by calculating the standard deviation of individual response (SD_IR_). They proposed that true variability exists when the SD_IR_ exceeds the smallest worthwhile change, defined as 0.2 times the standard deviation of a control group. Using this criterion, they confirmed significant interindividual variation in the adaptive increases of skeletal muscle citrate synthase activity and capillary density after 4 weeks of cycling exercise training. Collectively, these studies confirm the existence of substantial biological diversity in response to external stimuli. Nevertheless, the underlying mechanisms that give rise to such interindividual variation remain largely elusive. This gap in knowledge may be attributed to the inherent complexity of the biological systems involved, which arises from their multifactorial and person-specific nature, posing a substantial challenge to identifying the determinants of individual responsiveness.

Recent advances in artificial intelligence (AI) have enabled in silico analyses to uncover complex patterns and high-dimensional relationships across biological datasets. In bioinformatics, deep learning models trained on genome-wide multi-omics data have been successfully applied to infer the regulatory logic of transcriptional control and to identify key molecular drivers of disease. For example, Xi et al.^6^ developed a neural network that integrates transcription factor expression with chromatin accessibility at cis-regulatory elements using a multi-head self-attention mechanism, enabling accurate prediction of gene expression and classification of cell states from single-cell multi-omics data, as well as the identification of candidate regulatory factors in type 2 diabetes. In single-cell and spatial omics, recent advances in AI-driven methods have established a range of graph- and generative-model approaches. Graph neural networks (GNNs) have been applied to quantify network rewiring across conditions, prioritize candidate regulators, and capture both short- and long-range cell–cell interactions in spatial transcriptomics^7,8^. Multi-view and path-based frameworks have enabled the integration of inter- and intra-cellular signaling, allowing gene-expression imputation and the inference of ligand–gene regulatory relationships at single-cell resolution^9^. In addition, graph-based models using single-cell ATAC-seq data have been developed to reconstruct higher-order 3D chromatin compartment structures, while dual-topology graph convolutional networks have improved unsupervised clustering of heterogeneous transcriptomes^10,11^. Beyond GNNs, diffusion-based generative models have been introduced to generate virtual transcriptomic profiles, offering applications in drug-response prediction^12^. Together, these advances illustrate how deep learning architectures are increasingly capable of modeling both the structural and functional variation underlying complex cellular systems. While these AI-based models provide significant insights into cellular-level variation and regulation, a deeper understanding of interindividual biological variations, particularly in response to physiological stimuli, requires a broader, macro-scale modeling approach. Such a model must integrate molecular networks and physiological parameters with environmental influences that encompass both biological and physiological factors, enabling individualized inference on a mechanistic basis.

To investigate the biological mechanisms underlying interindividual variation, it is essential to move beyond static representations of biological systems and consider the dynamic, context-dependent relationships among molecular and physiological factors. While canonical pathway databases such as KEGG have been widely used to interpret high-throughput data by identifying enriched signaling pathways, these static maps often fail to reconcile inconsistencies observed in empirical studies. For instance, experimentally validated changes in gene expression or protein abundance may not align with expected pairwise interactions suggested by public databases. In several cases, only one component of a canonical interaction pair shows significant regulation, whereas the other remains unchanged, suggesting that the activation of biological pathways is conditional and context-specific. Such discrepancies likely stem from heterogeneity in experimental conditions, including differences in tissue types, environmental stimuli, or subject-specific physiological states. To address these limitations, we propose that a dynamic biological network, in which multiple, potentially conflicting factor relationships inferred from diverse experimental contexts are allowed to coexist and interact, is required for accurately modeling individual-specific biological responses. In this study, we present a novel deep learning architecture based on a GNN, designed to infer individualized mechanisms of "bioreaction-variation" — a term that refers to the interindividual variation in biological responses to physiological stimuli. Our GNN framework integrates both molecular-level and physiological-level nodes and is capable of identifying the most plausible mechanistic pathways that explain observed data under specific experimental contexts. Furthermore, the model is also designed to be implementable at a laboratory scale, with a minimized structure that facilitates targeted inference for specific tissues or physiological functions. This design enables context-aware inference of individualized biological networks, thereby offering a scalable and mechanistically interpretable approach for elucidating complex interindividual variation.

## Methods

### Overview of bioreaction-variation network

The present study introduces a bioreaction-variation network constructed using a GNN framework designed to capture the relationships between experimental models and corresponding physiological or biological parameters, as well as the interactions among these parameters. The GNN architecture comprises two core components: 1) a Model-to-Target interaction layer, which learns the associations between experimental conditions and observed outcomes, and 2) a Target-to-Target interaction layer, which models the interrelationships among the measured parameters themselves. All training data were curated from published experimental studies, with each parameter represented as a change or difference induced by a specific experimental intervention. This structure enables the model, upon input of new experimental data, including detailed conditions and observed parameter changes, to infer relevant biological pathways that mechanistically link experimental conditions and observed physiological responses, enabling individualized interpretation based on patterns learned from previously published studies.

### Training data collection

A total of 65,096 published studies were obtained from PubMed Central. These articles were identified by first searching PubMed with the keyword “skeletal muscle”, and subsequently filtering for those available as free full text in PubMed Central. The main texts of the selected articles were processed using the GPT-4o-mini model to extract relevant experimental contexts. Each study was summarized into a structured format following a fixed JSON schema (Table 1). Each JSON entry represented a discrete experimental unit, capturing the details of the experimental model, the physiological or molecular parameters analyzed, and the direction of change.

**Table 1:** Attributes and descriptions for data collection.

Attribute	Description
PMID	Article ID provided in PubMed
Species	The species used in the experiment using the common name
Age	The age or weeks old at the time of the experiment
Sex	The biological sex of the subject
Biosample_main	The type of tissue or cells used in the experiment
Biosample_detail	Additional details about the biosample, such as tissue or cell name
Experiment_type	The experimental level, e.g., in vivo, in vitro, ex vivo, case study
Model_main	The main physiological stimulus used in the experiment
Model_detail1	The first level of details, such as specific stimulus type
Model_detail2	Further details, such as intensity, dosage, duration, or frequency
Model_detail3	Additional specific experimental conditions if necessary
Timepoint	Chronological point at which the sample was collected for analysis
Targets	
Target	The specific name of the measured factor
Molecule_type	Specify the type of molecule, e.g., protein, mRNA
Analysis_main	The primary measurement method, e.g., western blotting, PCR
Analysis_detail	Additional details about the measurement method
Relation	The type of observed change, e.g., increase, decrease
Change	The degree of change in percentage or fold change
Significance	The statistical significance using p-values
Control	The control group used for comparison in “relation”

Descriptions shown in this table are excerpts from the actual prompts used in the GPT-4o-mini model for attribute formatting. Note: The attribute was filled by “none”, if applicable answer did not meet in the article.

### Input data

The input data refer to practical datasets used to perform inference with the trained model. In this study, model performance was initially validated using virtual input data constructed to simulate biologically plausible scenarios (see Supplementary data.zip online). Additionally, inference was conducted using real experimental data (see Supplementary data.zip online), derived from RNA sequencing results of individual mouse skeletal muscle samples. These datasets were originally reported in our previous publication^13^ and have been deposited on the official journal website associated with that article. To assess interindividual variability, fold changes in gene expression between exercised and non-exercised control mice were calculated across all possible pairwise combinations of individuals. For example, expression profiles of exercise #1 were compared independently to those of control #1, #2, and #3. Genes exhibiting differential responses to exercise were selected based on the following criterion: at least one exercised mouse showed an average fold change in gene expression greater than twofold when compared to all control mice, while the remaining exercised mice exhibited either changes below the twofold threshold or responses in the opposite direction. A total of 15 genes met this criterion and were used for individualized inference in downstream analyses (see Supplementary Fig. S1 online).

### Embedding

To encode experimental context into vector representations, BioBERT (ver. 1.1)^14^ was used to tokenize the descriptive elements of each study. Graph construction was performed using PyTorch Geometric (ver. 2.6.1)^15^, in which nodes were defined based on the “model main” categories (model nodes) and individual “target” entries (target nodes) extracted from each publication. To support node-level learning, node embeddings were also aggregated into mean vectors representing the average features across identical model or target nodes, thereby capturing generalizable node representations across the dataset. Further details on the embedding procedures are provided in the Supplementary Methods online.

### GNN model learning

**Fig. 1:**
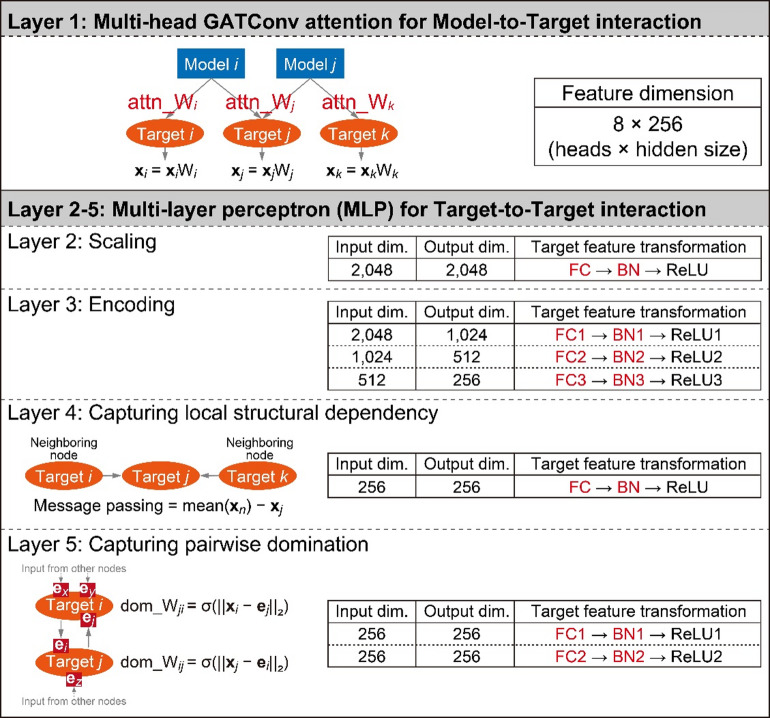
GNN model training overview. The graph neural network (GNN) model was trained to capture both Model-to-Target and Target-to-Target interactions through a structured, layer-wise architecture. Layer 1 employed a multi-head Graph Attention Convolution (GATConv) to compute attention weights (attn_W) for each Target node based on associated Model nodes. These attention weights are learnable parameters that allow the model to assign dynamic importance to each model-target interaction. The mechanism operated across 8 attention heads, each with a 256-dimensional hidden space. Here, **x** denotes the 768-dimensional feature vector of a Target node, aggregated as the mean of all features assigned to the same target entity. Layers 2 to 5 focused on Target-to-Target interactions through a multi-layer perceptron (MLP). Layer 2 applied a fully connected (FC) transformation with batch normalization (BN) and ReLU activation, preserving the feature dimension at 2,048. Layer 3 sequentially encoded the feature vector via three FC-BN-ReLU blocks, progressively reducing the dimensionality from 2,048 to 256. Layer 4 captured local structural dependencies by computing message passing, defined as the difference between the mean feature vector of neighboring nodes (**x**_*n*_) and the current node’s feature (**x**_*j*_). This layer also included an FC-BN-ReLU block. Layer 5 inferred pairwise domination between connected Target nodes. The domination weight (dom_W) was calculated as the sigmoid of the feature distance between the mean feature of a target node (**x**_*i*_) and the incoming feature vector from another node (**e**_*j*_). Two FC-BN-ReLU blocks were used in this layer as well. All learnable parameters including FC, BN, and attn_W are shown in red.

GNN training was conducted using a five-layer architecture (Fig. 1). Full implementation details, including preprocessing scripts and model training code, are available via the GitHub repository (https://github.com/fumikawano-lab/Bioreaction-Variation-Network). To capture Model-to-Target interactions, the first layer employed a multi-head Graph Attention Convolution (GATConv) mechanism^16,17^ to compute attention weights (attn_W) for each target node. Model and target features, initially encoded as 768-dimensional BioBERT-based embeddings, were linearly transformed into 2,048-dimensional representations composed of 8 attention heads with 256 hidden dimensions per head.

1 $${\widetilde{\mathbf{m}}}_{i}={\text{BN}}_{m}\left({\mathbf{W}}_{m}{\mathbf{m}}_{i}\right), {\widetilde{\mathbf{t}}}_{j}={\text{BN}}_{t}\left({\mathbf{W}}_{t}{\mathbf{t}}_{j}\right),$$

where ${\mathbf{m}}_{i}\in {\mathbb{R}}^{768}$ denotes the feature of a model node *i*, and ${\mathbf{t}}_{j}\in {\mathbb{R}}^{768}$ denotes the feature of a target node *j*. After reshaping, each is split into *H* attention heads of dimension *d*.

2 $${\mathbf{u}}_{ij}={\mathbf{e}}_{ij}[1:768]-{\mathbf{e}}_{ij}[769:1536], {\mathbf{a}}_{ij}={\mathbf{W}}_{e}{\mathbf{u}}_{ij},$$

where ${\mathbf{e}}_{ij}\in {\mathbb{R}}^{1536}$ denotes the edge attribute vector between model node *i* and target node *j*.

3 $$\begin{gathered} \alpha_{ij}^{{\left( {h,m \to t} \right)}} = \frac{{{\text{exp }}\left( {{\text{PReLU }}\left( {\left\langle {{\mathbf{m}}_{i}^{\left( h \right)} ,{\mathbf{a}}_{ij}^{\left( h \right)} } \right\rangle } \right)/\tau_{mt} } \right)}}{{\sum_{{h^{\prime}}} {\text{exp }}\left( {{\text{PReLU }}\left( {\left\langle {{\mathbf{m}}_{i}^{{\left( {h^{\prime}} \right)}} ,{\mathbf{a}}_{ij}^{{\left( {h^{\prime}} \right)}} } \right\rangle } \right)/\tau_{mt} } \right)}}, \hfill \\ \alpha_{ij}^{{\left( {h,m \to n} \right)}} = \frac{{{\text{exp }}\left( {{\text{PReLU }}\left( {\left\langle {{\mathbf{m}}_{i}^{\left( h \right)} ,{\mathbf{a}}_{ij}^{\left( h \right)} } \right\rangle } \right)/\tau_{mn} } \right)}}{{\sum_{{h^{\prime}}} {\text{exp }}\left( {{\text{PReLU }}\left( {\left\langle {{\mathbf{m}}_{i}^{{\left( {h^{\prime}} \right)}} ,{\mathbf{a}}_{ij}^{{\left( {h^{\prime}} \right)}} } \right\rangle } \right)/\tau_{mn} } \right)}}, \hfill \\ \alpha_{ij}^{{\left( {h,t} \right)}} = \frac{{{\text{exp }}\left( {{\text{PReLU }}\left( {\left\langle {{\mathbf{t}}_{j}^{\left( h \right)} ,{\mathbf{a}}_{ij}^{\left( h \right)} } \right\rangle } \right)/\tau_{t} } \right)}}{{\sum_{{h^{\prime}}} {\text{exp }}\left( {{\text{PReLU }}\left( {\left\langle {{\mathbf{t}}_{j}^{{\left( {h^{\prime}} \right)}} ,{\mathbf{a}}_{ij}^{{\left( {h^{\prime}} \right)}} } \right\rangle } \right)/\tau_{t} } \right)}}. \hfill \\ \end{gathered}$$

Three types of attention coefficients are computed. The model-to-target attention (m → t) quantifies the contribution of a model node to a target node via edge attributes. The model-to-target feature reinforcement (m → n) indicates how strongly a model node should amplify the original feature of the target node. The target self-attention (t) captures the degree to which the target node’s own feature is emphasized relative to the edge attributes.

4 $$\begin{gathered} {\mathbf{u}}_{ij}^{\left( h \right)} = \alpha_{ij}^{{\left( {h,m \to t} \right)}} { }{\mathbf{a}}_{ij}^{\left( h \right)} + \alpha_{ij}^{{\left( {h,m \to n} \right)}} { }{\mathbf{t}}_{j}^{\left( h \right)} + \alpha_{ij}^{{\left( {h,t} \right)}} { }{\mathbf{t}}_{j}^{\left( h \right)} , \hfill \\ {\mathbf{z}}_{ij} = \left[ {{ }{\mathbf{u}}_{ij}^{\left( 1 \right)} ;{ }{\mathbf{u}}_{ij}^{\left( 2 \right)} ;{ } \ldots ;{ }{\mathbf{u}}_{ij}^{\left( H \right)} { }} \right] \in {\mathbb{R}}^{{Hd_{h} }} . \hfill \\ \end{gathered}$$

Each edge-level message ${\mathbf{z}}_{ij}$ is formed by, for every head *h*, taking a weighted sum of the projected target feature and the edge attribute using the three attention coefficients, and then concatenating the *H* head-wise results.

5 $${\mathbf{x}}_{j}^{(1)}=\frac{1}{|{\mathcal{E}}_{MT}(j)|}\sum_{(i,j) \in {\mathcal{E}}_{MT}(j)} {\mathbf{z}}_{ij}.$$

For each target node *j*, the updated node feature ${x}_{j}^{(1)}$ is the simple mean of the edge-level messages ${z}_{ij}$ over all incoming edges $(i,j)\in {\mathcal{E}}_{MT}$.

Subsequent layers (Layers 2–5) implemented a multi-layer perceptron (MLP) to capture Target-to-Target interactions.

6a $${\mathbf{x}}_{j}^{(2)}=\text{ReLU }({\text{BN}}_{1}^{(2)} ({\mathbf{W}}_{1}^{(2)} {\mathbf{x}}_{j}^{(1)})), {\mathbf{W}}_{1}^{(2)}\in {\mathbb{R}}^{2048\times 2048}.$$

The second layer scales all target node features while preserving their dimensionality, ensuring consistent scaling across nodes without altering the representation size.

6b $$\begin{gathered} {\mathbf{h}}_{j}^{{\left( {3,1} \right)}} = {\text{ReLU }}\left( {{\text{BN}}_{1}^{\left( 3 \right)} { }\left( {{\mathbf{W}}_{1}^{\left( 3 \right)} { }{\mathbf{x}}_{j}^{\left( 2 \right)} } \right)} \right),{ }{\mathbf{W}}_{1}^{\left( 3 \right)} \in {\mathbb{R}}^{1024 \times 2048} , \hfill \\ {\mathbf{h}}_{j}^{{\left( {3,2} \right)}} = {\text{ReLU }}\left( {{\text{BN}}_{2}^{\left( 3 \right)} { }\left( {{\mathbf{W}}_{2}^{\left( 3 \right)} { }{\mathbf{h}}_{j}^{{\left( {3,1} \right)}} } \right)} \right),{ }{\mathbf{W}}_{2}^{\left( 3 \right)} \in {\mathbb{R}}^{512 \times 1024} , \hfill \\ {\mathbf{X}}_{j} = {\text{ReLU }}\left( {{\text{BN}}_{3}^{\left( 3 \right)} { }\left( {{\mathbf{W}}_{3}^{\left( 3 \right)} { }{\mathbf{h}}_{j}^{{\left( {3,2} \right)}} } \right)} \right),{ }{\mathbf{W}}_{3}^{\left( 3 \right)} \in {\mathbb{R}}^{256 \times 512} . \hfill \\ \end{gathered}$$

The third layer encodes the feature stepwise from 2,048 to 256 dimensions, producing the final representation ${\mathbf{X}}_{j}$ used in subsequent layers.

The fourth layer was designed to model local topological structure via message passing. Message passing in the training process was defined as the difference between a given target node’s feature and the average feature of its input neighbors, as derived from the original training data.

7 $$\begin{gathered} \varvec{\mu }_{j} = \frac{1}{{\left| {{\mathcal{N}}_{{TT}} \left( j \right)} \right|}}\sum\limits_{{i \in {\mathcal{N}}_{{TT}} \left( j \right)}} {{\mathbf{X}}_{i} } {\text{, }}\Delta {\mathbf{x}}_{j} = {\text{ReLU }}\left( {{\text{BN}}_{x} \left( {{\mathbf{W}}_{x} \left( {\varvec{\mu }_{j} - {\mathbf{X}}_{j} } \right)} \right)} \right), \hfill \\ {\mathbf{M}}_{j} = {\mathbf{X}}_{j} + \Delta {\mathbf{x}}_{j} . \hfill \\ \end{gathered}$$

Here, ${\mathcal{N}}_{TT}(j)$ denotes the set of target nodes directly connected to target node *j* within the target–target graph. The term ${{\varvec{\mu}}}_{j}$ represents the mean feature vector of the neighbors of *j*, and $\Delta {\mathbf{x}}_{j}$ is the predicted message passing update, obtained as the difference between the neighbor mean and the node’s own representation after transformation and normalization. Finally, ${\mathbf{M}}_{j}$ is defined as the updated representation of node *j*, where ${\mathbf{x}}_{j}$ is augmented with the message passing it would receive if the entire network were activated.

8 $$\begin{gathered} \overline{\mathbf{e}}_j= \frac{1}{\lvert \mathcal{N}(j)\rvert}\,\sum\limits_{i \in \mathcal{N}(j)} \big(\mathbf{e}_{ij}[1{:}768] - \mathbf{e}_{ij}[769{:}1536]\big),\hfill \\ \widehat{{{{\Delta }}{\mathbf{x}}}}_{j} = {\text{ReLU }}\left( {{\text{Norm}}\left( {{\mathbf{W}}_{e}^{{\left( {{\text{orth}}} \right)}} \overset{\lower0.5em\hbox{$\smash{\scriptscriptstyle\leftharpoonup}$}}{{\mathbf{e}}} _{j} } \right)} \right). \hfill \\ \end{gathered}$$

The teacher signal ${\widehat{\Delta \mathbf{x}}}_{j}$ is derived directly from edge attributes. Specifically, the averaged edge difference vector ${\overline{\mathbf{e}}}_{j}$ is linearly transformed using a weight matrix ${\mathbf{W}}_{e}^{(\text{orth})}$, which is initialized as an orthogonal matrix and kept fixed during training. This transformation also projects the teacher signal into a 256-dimensional space, thereby matching the dimensionality of the model-predicted update $\Delta {\mathbf{x}}_{j}$. This design ensures that the teacher signal provides a stable and unbiased reference for message passing, independent of the model’s parameter updates.

The fifth layer modeled pairwise dominance among target nodes using a domination weight.

9 $${d}_{i\to j}=\| {\mathbf{A}}_{j}-{\mathbf{A}}_{i\to j}{\| }_{2}, {W}_{i\to j}=\sigma \left(\beta ({d}_{i\to j}-{d}_{0})\right).$$

Here, ${W}_{i\to j}$ denotes the domination weight, representing how strongly the feature representation of node *j* depends on the input from node *i*.

10 $$\begin{gathered} \overline{W}_{j} = \frac{1}{{\left| {{\mathcal{N}}\left( j \right)} \right|}}{ }\mathop \sum \limits_{{i \in {\mathcal{N}}\left( j \right)}} W_{i \to j} , \hfill \\ {\mathbf{D}}_{j}^{{{\text{teacher}}}} = {\text{ReLU }}\left( {{\text{Std}}\left( {{\mathbf{W}}_{d}^{{\left( {{\text{orth}}} \right)}} \left( {\overline{W}_{j} \cdot {\mathbf{A}}_{j} } \right)} \right)} \right). \hfill \\ \end{gathered}$$

For each target node *j*, the domination weights from all connected neighbors $i\in \mathcal{N}(j)$ are computed and averaged to form the aggregated weight ${\overline{W}}_{j}$. The resulting ${\mathbf{D}}_{j}^{\text{teacher}}$ provides the teacher signal for domination, serving as the fixed reference in the supervised learning of dominance.

11 $${\mathbf{D}}_{j}^{\text{out}}=\text{ReLU }\left({\text{BN}}_{2} \left({\mathbf{W}}_{2}\text{ ReLU}({\text{BN}}_{1}({\mathbf{W}}_{1}{\mathbf{M}}_{j}))\right)\right).$$

The domination output is the final model prediction of the GNN model learning, obtained by transforming the updated node representation. This vector represents the learned dominance distribution over the connected nodes.

12a $$\text{Dom}(i\to j):=({\mathbf{D}}_{j}^{\text{out}}{)}_{i}, i\in \mathcal{N}(j).$$

12b $$i\succ j {\text{ iff }} ({\mathbf{D}}_{j}^{\text{out}}{)}_{i}>({\mathbf{D}}_{j}^{\text{out}}{)}_{j}.$$

Here, the pairwise domination relation $\text{Dom}(i\to j)$ is defined as the *i*-th component of the model-predicted domination output vector for the target node *j*, ${\mathbf{D}}_{j}^{\text{out}}$. In practice, for each target node *j*, the domination scores for all connected neighbors $i\in \mathcal{N}(j)$ are computed and employed during network exploration; an oriented edge $i\to j$ is preferred when $\text{Dom}(i\to j)$ exceeds $\text{Dom}(j\to i)$.

The training loss was computed as the mean squared error (MSE) between the model-predicted outputs and the corresponding teacher signals: the message passing updates in the fourth layer (message passing loss) and the final dominance vectors in the fifth layer (domination loss).

13 $${\mathcal{L}}_{\text{message}}=\frac{1}{|\mathcal{T}|} \sum_{j\in \mathcal{T}} \|\Delta {\mathbf{x}}_{j}-{\widehat{\Delta \mathbf{x}}}_{j}{\| }_{2}^{2}.$$

14 $${\mathcal{L}}_{\text{dom}}=\frac{1}{|\mathcal{T}|} \sum_{j\in \mathcal{T}} \| {\mathbf{D}}_{j}^{\text{out}}-{\mathbf{D}}_{j}^{\text{teacher}}{\| }_{2}^{2}.$$

15 $${\mathcal{L}}_{\text{total}}=2.0 {\mathcal{L}}_{\text{message}}+0.1 {\mathcal{L}}_{\text{dom}}.$$

The learning was formulated under the assumption that the entire network is activated, such that every node receives input signals as if all physiological stimuli were simultaneously present. Under this condition, the message passing loss evaluates the accuracy of the predicted influence from proximal biological factors, which enables the model to reconstruct local subgraph structures. Importantly, when the whole network is assumed to be activated, the node features obtained after message passing can be interpreted as reflecting the inherent positioning of each node within the biological system. The domination loss further evaluates the consistency of edge directionality, providing a quantitative criterion for embedding competitive up- and downstream relationships among parameters that were simultaneously analyzed within individual studies. By jointly optimizing these two losses, the model learns node features and directional connectivity in a manner that reflects their functional positions in the global biological network.

Model optimization was performed using Adam optimizer^18^ with a learning rate of 0.001, and backpropagation was carried out over 50 or 200 total epochs. To mitigate gradient bias, total loss was computed as a weighted sum of the two loss components: message passing loss × 2.0 and domination loss × 0.1 (Eq. 15). Final trained models are publicly available at Google Cloud Storage (https://storage.googleapis.com/skeletal_muscle/sm_v1/gnn_model/gnn_model_final.pt for 200-epoch model and gnn_model_final_50epoch.pt for 50-epoch model).

### Network inference

**Fig. 2:**
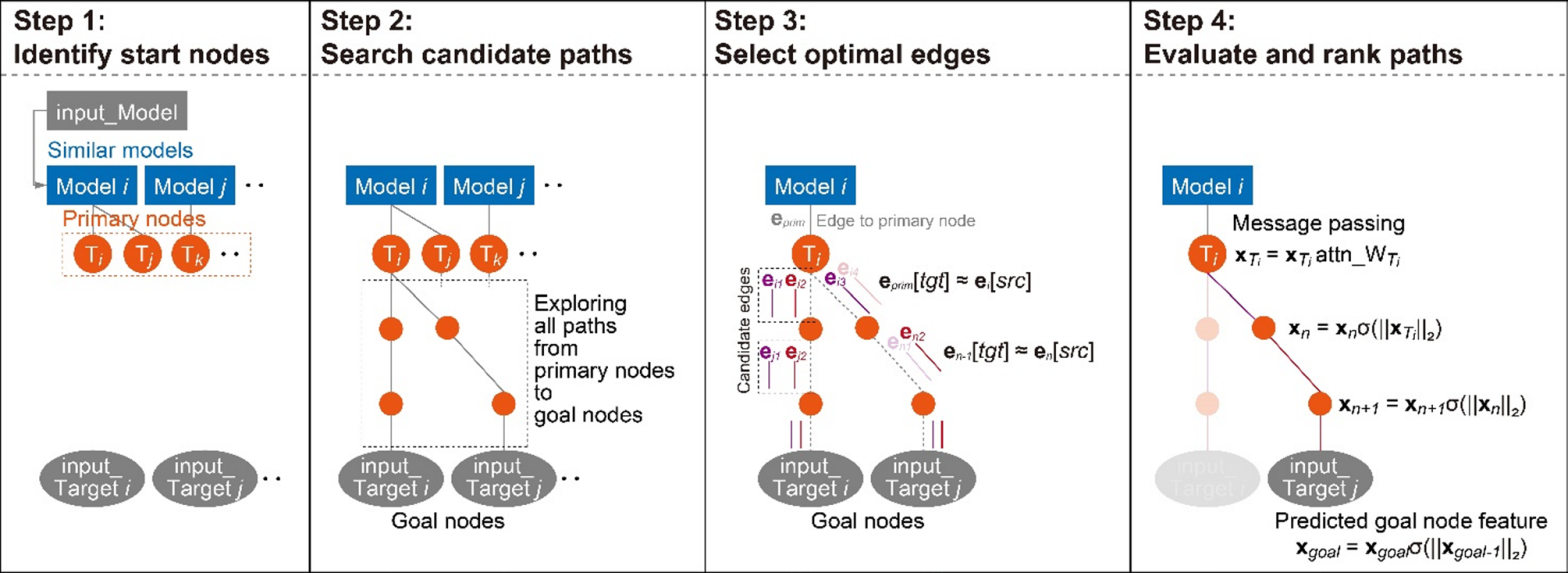
Network inference overview. Step 1: The top five models in the training dataset most similar to the input model (based on model feature similarity) were identified. The target nodes directly connected to these models were then designated as primary nodes, serving as the starting points for network inference. Step 2: Using the trained GNN model, all candidate paths were explored from the primary nodes (start nodes) toward the input target nodes, which served as the goal nodes. Step 3: For each source–target node pair along the paths, the optimal edge was selected from a pool of candidate edges. The edge selection criterion required that the source node of the current edge closely matched the target node of the previous edge in the sequence (i.e., **e**_*n-1*_[tgt] ≈ **e**_*n*_[src]), thereby ensuring topological continuity toward the goal node. Step 4: Message passing was applied along each candidate path. For the first target node, the attention weight (attn_W) from the connected model node was applied. Subsequent nodes received propagated signals scaled by the sigmoid-transformed Euclidean norm of the previous node’s feature (i.e., σ(║**x**_*n*_║_2_)). The predicted feature of each goal node (**x**_*goal*_) was obtained at the end of the path. The best combination of paths was selected for each goal node by minimizing the Euclidean distance between the predicted node feature and the actual input feature across all possible path combinations.

To infer individual-specific hidden pathways, network traversal was performed based on user-provided input data comprising experimental model metadata and observed target parameters. These inputs, formatted consistently with the training data structure, were embedded into 768-dimensional feature vectors using BioBERT, following the same preprocessing pipeline used during model training. To initiate the inference process, cosine similarity was computed between the input model vector and all model embeddings in the training dataset. The top five most similar model nodes were selected, and their directly connected target nodes were identified as primary targets, serving as start nodes for subsequent traversal (Step 1 in Fig. 2). The aim was to reconstruct viable paths from these start nodes to goal nodes, which corresponded to the target features present in the user-provided input. In Step 2, all possible directed paths between the identified start and goal nodes were enumerated based on the GNN-learned edge connectivity between target nodes. This yielded a candidate path space representing potential mechanistic routes through the network. In Step 3, path refinement was performed by selecting the most contextually relevant edges at each intermediate node. When multiple edges existed between the same node pair (i.e., same source and target nodes but differing feature contexts), the edge whose source feature was most similar to the preceding target node, as determined by cosine similarity, was selected. This ensured feature continuity across the reconstructed path.

In Step 4, message passing was performed from each primary node through candidate paths, and the propagated features at the goal nodes were compared with the input goal features. The subsets of paths that minimized this discrepancy were iteratively selected using a greedy beam-style procedure. The final output corresponds to the best combination of paths whose averaged predictions most accurately reproduced the input goal features. Mathematically, this procedure can be described as follows.

For each primary node ${T}_{i}$ with feature vector ${\mathbf{x}}_{{T}_{i}}\in {\mathbb{R}}^{d}$, an attention-based scalar weight was applied to initialize the node representation:

16 $${\hat{\mathbf{x}}}_{{T_{i} }} = \alpha_{{T_{i} }} { }{\mathbf{x}}_{{T_{i} }} ,{ }\alpha_{{T_{i} }} \in {\mathbb{R}}.$$

Here, ${\alpha }_{{T}_{i}}\in {\mathbb{R}}$ denotes the attention-derived weight, and ${\hat{\mathbf{x}}}_{{T_{i} }}$ represents the adjusted primary feature used to start the propagation.

Message passing along a candidate path $p:{v}_{0}\to {v}_{1}\to \cdots \to {v}_{L}({v}_{0}={T}_{i}, \, {v}_{L}=g)$ was implemented as a multiplicative update. At each step $l=1,\dots ,L$, the node feature was updated according to

17 $$\begin{gathered} {\mathbf{x}}_{{v_{l} }}^{\left( l \right)} = s_{l} { }{\mathbf{t}}_{{e_{l} }} , \hfill \\ s_{l} = c{ }\sigma { }\left( {\alpha \left( {\left\| {{\mathbf{x}}_{{v_{l - 1} }}^{{\left( {l - 1} \right)}} } \right\|_{2} - \beta } \right)} \right). \hfill \\ \end{gathered}$$

In this formulation, ${e}_{l}=({v}_{l-1} \to {v}_{l},{m}_{l})$ is the selected edge, ${\mathbf{t}}_{{e}_{l}}\in {\mathbb{R}}^{d}$ is its target-side feature, and ${s}_{l}$ is a scalar intensity determined by the norm of the preceding node feature. The constants c > 0, α > 0, and $\beta \in {\mathbb{R}}$ regulate the scaling and nonlinearity applied through the sigmoid function.

The prediction obtained at the goal node *g* from a single path *p* was expressed as

18 $${\mathbf{\hat{x}}}_{g} \left( p \right) = {\mathbf{x}}_{{v_{L} }}^{{\left( L \right)}} .$$

This corresponds to the propagated feature obtained after traversing the entire path.

During the best-combination search, subsets of candidate paths were iteratively selected for each goal node. For a subset ${S}_{g}\subseteq {\mathcal{P}}_{g}$, the aggregated prediction was defined as

19 $${\mathbf{\bar{x}}}_{g} \left( {S_{g} } \right) = \frac{1}{{\left| {S_{g} } \right|}}\sum\limits_{{p \in S_{g} }} {{\mathbf{\hat{x}}}_{g} \left( p \right)} .$$

This aggregation involves only the paths in the selected subset ${S}_{g}$ and serves as the basis for evaluating agreement with the input goal feature.

The discrepancy between the aggregated predictions and the input goal features was quantified by the loss

20 $${\mathcal{L}}\left( S \right) = \mathop \sum \limits_{{g \in G_{{{\text{goal}}}} }} \left\| {{\overline{\mathbf{x}}}_{g} \left( {S_{g} } \right) - {\mathbf{y}}_{g} } \right\|_{2}^{2} ,$$

where ${\mathbf{y}}_{g}$ is the input goal feature and $S=\{{S}_{g}{\}}_{g\in {G}_{\text{goal}}}$ represents the collection of selected subsets across all goals.

The subsets were updated by adding the candidate path *p* that minimized the squared error of the already-selected paths $q\in {S}_{g}^{(t)}$ augmented with *p*, at each iteration:

21 $$\begin{gathered} p^{{ \star }} = \operatorname{arg} \min\limits_{\,p \in \mathcal{P}_{g} \setminus S_{g}^{(t)}} \left\| {\frac{1}{{\left| {S_{g}^{\left( t \right)} } \right| + 1}}\left( {\mathop \sum \limits_{{q \in S_{g}^{\left( t \right)} }} {\hat{\mathbf{x}}}_{g} \left( q \right) + {\hat{\mathbf{x}}}_{g} \left( p \right)} \right) - {\mathbf{y}}_{g} } \right\|_{2}^{2} , \hfill \\ S_{g}^{{\left( {t + 1} \right)}} = S_{g}^{\left( t \right)} \cup \left\{ {p^{{ \star }} } \right\}. \hfill \\ \end{gathered}$$

This procedure was repeated until no further improvement was achieved or the maximum beam width ${K}_{g}$ was reached. The final subsets for each goal, denoted ${S}_{g}^{(\text{final})}$, were collected into

22 $${S}^{\star }=\{{S}_{g}^{(\text{final})}\mid g\in {G}_{\text{goal}}\},$$

which was defined as the best combination. This output represents the path subsets whose averaged predictions most accurately reproduced the input goal features.

Steps 3 and 4 were embedded in a genetic algorithm framework using the DEAP library^19^. Each candidate path generated in Step 2 was treated as an individual. Fitness was evaluated as a weighted combination of loss and diversity, the latter reflecting the uniqueness of nodes and path topology. Path selection employed the Elitist Non-dominated Sorting Genetic Algorithm (NSGA-II), with mutation applied to 20% of individuals per generation. Mutation involved random substitution of an edge within a path, followed by structural repair using the same edge selection strategy described in Step 2 and 3. If the mutation resulted in higher loss, the original path configuration was retained. Given that the initial path candidates were exhaustively constructed and optimized during Steps 2 and 3, the evolutionary loop was iterated twice, solely to confirm that no further improvement in fitness metrics could be achieved. All source code and execution details for the network inference procedure are available at the associated GitHub repository (https://github.com/fumikawano-lab/Bioreaction-Variation-Network).

### Analysis of individualized networks

To identify individual-specific pathways, inferred GNN outputs for each exercised mouse (n = 3) were compared against all non-exercised controls (n = 3). For each exercised mouse, individual networks were constructed by aggregating all predicted paths obtained from pairwise comparisons with each control mouse. A common network was then defined as the set of paths shared across all three individual networks, while unique networks were obtained by subtracting the common network from each individual’s network. To assess overall reconstruction accuracy, message passing loss was calculated for each goal node and averaged across the network. In addition, among non-primary and non-goal nodes, the most frequently occurring source node across all inferred networks was identified. For this node, the contribution of each connected target node was assessed by referencing the message passing loss of the specific path (i.e., evolutionary algorithm-derived individual) in which the corresponding edge was included. This provided a direct measure of each target node’s involvement in the reconstruction of input-derived goal node features within individualized networks.

## Results

### Overview of training data

The training graph was constructed from a total of 27,155 model nodes and 84,723 target nodes. These nodes were interconnected by 383,225 model-to-target edges and 2,475,502 target-to-target edges. Node frequency distributions are summarized in the Supplementary data.xlsx online. Briefly, the most frequently represented experimental models included exercise, high-fat diet feeding, sarcopenia, aging, muscle injury, electrical stimulation, type 2 diabetes, Duchenne muscular dystrophy, diabetes, and isometric contraction. Frequently analyzed target nodes encompassed IL-6, myogenin, MyoD, TNFα, PGC-1α, creatine kinase, insulin, myosin heavy chain, MuRF1, and atrogin1.

During model training, the domination loss decreased more rapidly than the message passing loss (see Supplementary Fig. S3 online), a trend that was accounted for by applying differential weighting to these two components in the total loss function. When equal weights were assigned, the message passing loss plateaued early in the training process, limiting further optimization. Although the domination loss approached a minimum around 50 epochs, the message passing loss continued to decline steadily, reducing the difference between the two losses from 0.269 at epoch 50 to 0.228 at epoch 200. These results indicate that training for 200 epochs did not result in overfitting and that both 50-epoch and 200-epoch models were retained for downstream inference to assess potential differences in generalization.

### Validation of model with virtual input data

**Fig. 3:**
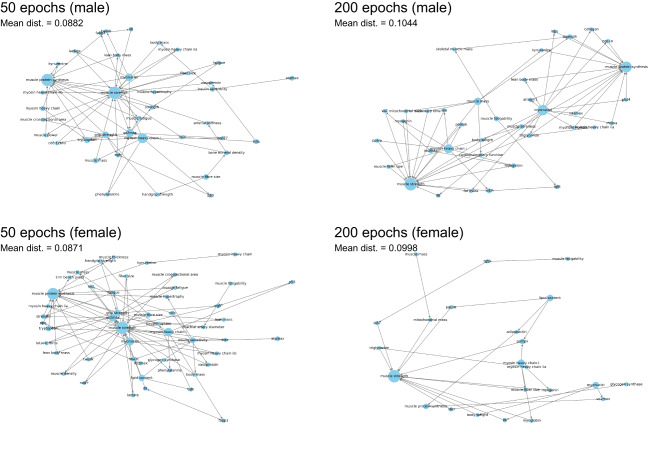
Model validation using virtual input data. Network graphs illustrate all inferred paths based on virtual input data in which the only differing attribute was the biological sex of the subjects. Model training was performed for either 50 or 200 epochs. The message passing loss was calculated for the best combination of inferred paths associated with each goal node, and these values were averaged to produce the mean distance (mean dist.).

To evaluate the inference performance of the trained GNN model, two types of virtual input datasets were constructed that differed only in the biological sex of the subjects, with all other experimental conditions held constant. In the networks inferred from the model trained over 50 epochs, key nodes such as myosin heavy chain I, myonuclei, and VO₂ max were frequently identified in both male and female datasets; however, the surrounding edge structures differed noticeably between sexes (Fig. 3). Similar nodes were also retrieved from the model trained over 200 epochs, but no clear improvement was observed in the mean distance (average message passing loss) compared to the 50-epoch model, suggesting that inference performance had already stabilized by that point.

### Network inference with real experimental data

**Fig. 4:**
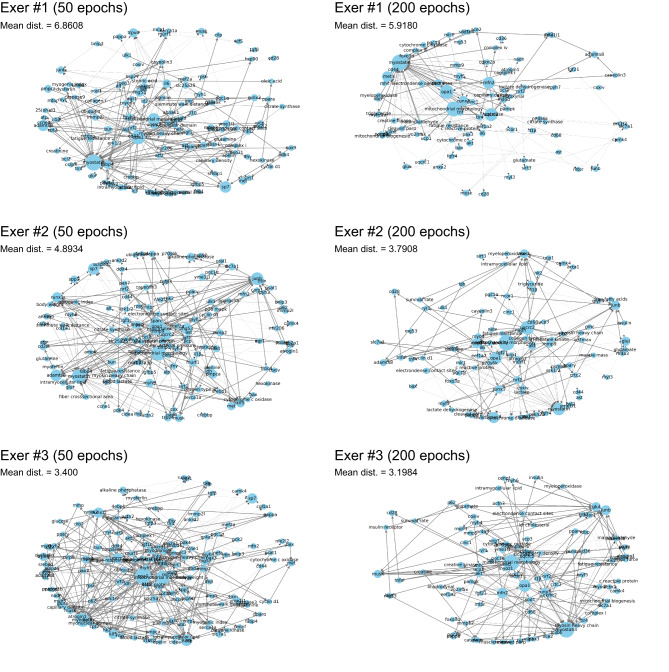
Network inference using real experimental data. Network graphs illustrate all inferred paths for each exercised mouse (Exer #1–3), based on reanalyzed RNA sequencing data from individual tibialis anterior muscle samples obtained in our previous study^13^. A single bout of treadmill running was conducted after a 4-week training protocol. Gene expression profiles were compared between three exercised mice and three non-exercised controls. See the Methods section for details on the individual comparisons. Model training was performed for either 50 or 200 epochs. The message passing loss was calculated for the best combination of inferred paths associated with each goal node, and these values were averaged to produce the mean distance (mean dist.).

To investigate individualized transcriptional responses to exercise, we reanalyzed skeletal muscle RNA sequencing data from exercised and non-exercised mice (n = 3 each) previously reported in our study^13^. Differentially expressed genes between exercised and non-exercised conditions were used to construct input features, which were then inferred through the trained GNN models (trained for either 50 or 200 epochs). Because the contextual input for the experimental model (e.g., exercise protocol, species, tissue) was identical across the three exercised mice, the same primary nodes were consistently identified. However, the inferred networks showed marked interindividual variation in the structure and composition of intermediate and goal nodes (Fig. 4). These differences were reflected in the mean message passing loss, which varied across individuals. Notably, inference using the 200-epoch trained model yielded lower average loss values in all individuals compared to the 50-epoch model, indicating improved network reconstruction performance.

**Fig. 5:**
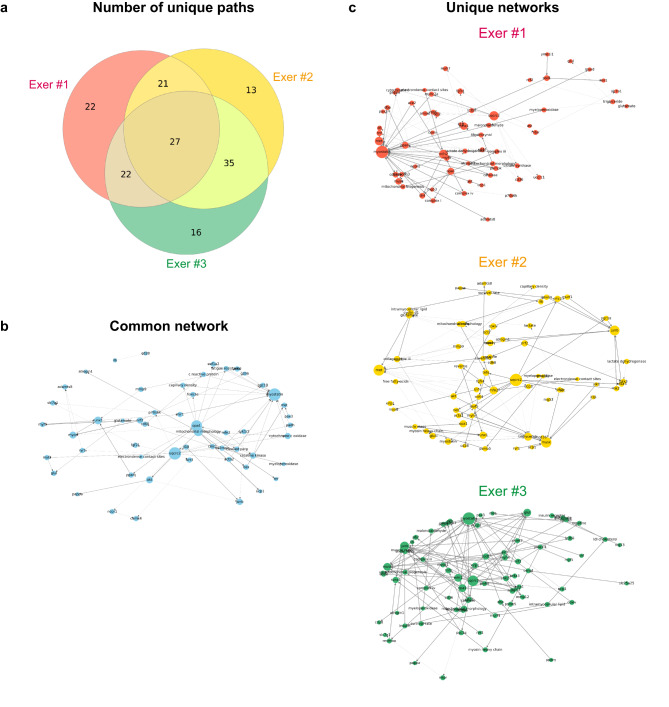
Extraction of common and individual-specific networks. (**a**) Venn diagram showing the number of shared and unique inferred paths among the three exercised mice (Exer #1–3). (**b**) The common network represents the overlapping paths found in all three individuals. (**c**) Unique networks for each mouse were extracted from the total inferred networks presented in Fig. 4 by identifying individual-specific paths.

**Fig. 6:**
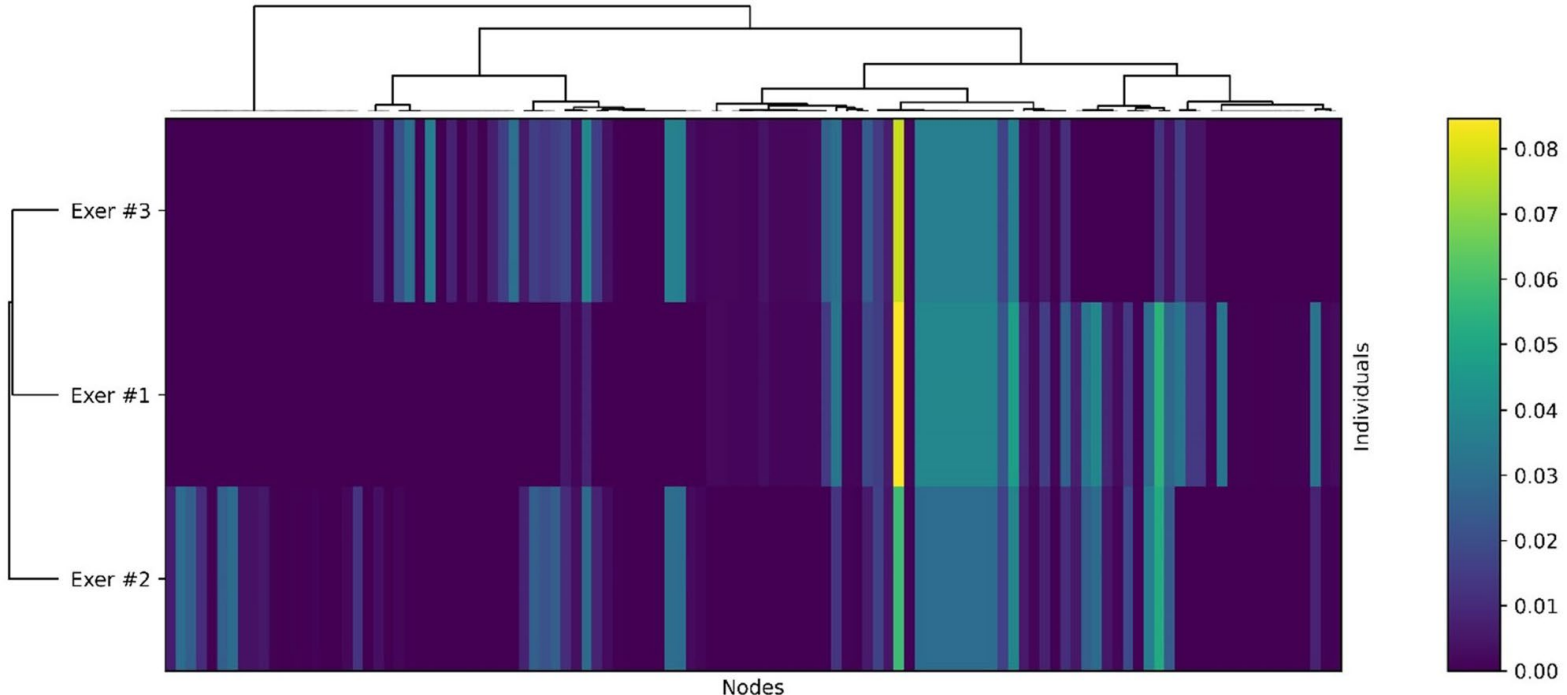
Clustering of node PageRank centralities across individuals. For each individual-specific network shown in Fig. 4, PageRank centrality was computed from path node sequences, with path weights determined by losses relative to the input-derived goal node features. Individuals were clustered by Jensen–Shannon divergence between their PageRank distributions (excluding the primary and goal nodes) using hierarchical clustering. Nodes absent from an individual’s network were assigned a centrality of zero. The three exercised mice (Exer #1–3) were each assigned to separate clusters, indicating mutually divergent network structures.

Given the superior performance of the 200-epoch model, we proceeded to identify common and unique network paths among individuals using this model. A path was defined as a complete sequence of connected nodes from a primary node to a goal node. A total of 27 paths were commonly found across all three individuals, involving key nodes such as AKT, creatine kinase, FOXO3A, mitochondrial morphology, OPA1, and UQCRC2 as intermediate nodes (Fig. 5a, b). Unique networks were obtained by subtracting this common network from each individual’s network (Fig. 5c). For example, Exer #1 exhibited 65 unique paths, of which 21 overlapped with Exer #2 and 22 with Exer #3 (Fig. 5a). Exer #2 and Exer #3 shared 35 paths (Fig. 5a), indicating that, among the three exercised mice analyzed, these two mice shared similar characteristics in the individual-specific regulatory mechanisms governing transcriptional responses to exercise. PageRank centrality was calculated to analyze the nodes structuring the GNN shown in Fig. 4 (see Fig. 6 and Supplementary data.xlsx online). OPA1 was the most frequently observed node across individuals. IL-6 and MFN2 were highly ranked in Exer #1 and Exer #2, whereas glutamate and PGC-1α showed higher centrality in Exer #3. COXIV was also frequently detected, being common to Exer #1 and Exer #3. Hierarchical clustering further revealed that these three individuals were differentially classified based on their centralities.

**Fig. 7:**
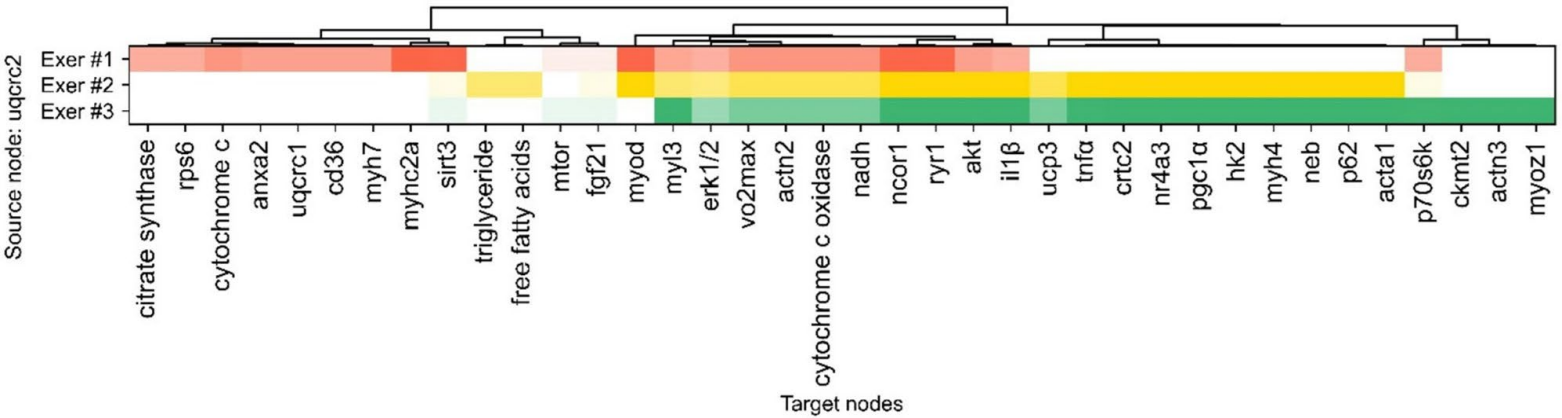
Variation of UQCRC2-associated edges. Heat map illustrating the target nodes connected to UQCRC2 across the three exercised mice (Exer #1–3). Targets were re-ordered by hierarchical clustering. Color intensity reflects the message passing loss of the path including the corresponding edge, with darker blocks indicating lower loss.

To further explore key regulatory factors potentially explaining interindividual variation, we focused on intermediate source nodes, excluding primary and goal nodes, and identified those with the highest frequency across all individuals. UQCRC2 emerged as the most recurrent source node in the inferred networks. This high recurrence indicated that UQCRC2 contributed most consistently to distinguishing the hidden network structures across individuals. Figure 7 illustrates the target nodes connected to UQCRC2 in each individual. UQCRC2 formed unique edges to citrate synthase, RPS6, cytochrome c, ANXA2, UQCRC1, CD36, MYH7, MYHC2A, and SIRT3 exclusively in Exer #1. While Exer #2 and Exer #3 shared several downstream nodes, edges from UQCRC2 to triglycerides and free fatty acids were specific to Exer #2, whereas P70S6K, CKMT2, ACTN3, and MYOZ1 were strongly associated with Exer #3. All edges identified from both common and unique paths are listed in the Supplementary data.xlsx online, along with the message passing loss of the paths in which each edge is included.

## Discussion

This study presents a GNN model capable of inferring latent networks that reflect individual-specific responses to physiological stimuli. The model was trained on a dataset constructed from published studies retrieved using the keyword “skeletal muscle”. As of July 2025, approximately 258,000 articles are indexed under this keyword, of which 104,000 are available as free full-text articles via PubMed Central. Our training dataset included 65,096 of these articles, covering more than one-fourth of the accessible literature on skeletal muscle.

The predominant experimental models extracted from these publications—such as exercise, high-fat diet, sarcopenia, aging, muscle injury, electrical stimulation, type 2 diabetes, and Duchenne muscular dystrophy—indicate that the dataset reflects biologically relevant contexts closely tied to skeletal muscle research. Accordingly, the extracted parameters and target nodes largely comprised skeletal muscle-related molecules and physiological outcomes. Notably, the model was designed to integrate both molecular and physiological parameters. Among the physiological parameters, non-muscle-related parameters such as VO_2_ max, blood glucose, and plasma hormone levels were also included, allowing the reconstruction of networks that reflect both intracellular signaling and systemic physiological outcomes. Rather than focusing solely on inferring molecular cascades, the model learned connections between parameters that frequently co-occur across diverse experimental contexts. This architecture enables the exploration of novel, individualized routes from experimental models to observed phenotypes. This capability is likely supported by the model’s global network structure, which connects parameters across distinct publications through shared nodes and feature similarities. As demonstrated by the inference with virtual input data (Fig. 3), even a single biological difference, such as sex, led to the identification of markedly distinct hidden networks. These results highlight the model’s ability to adaptively explore individualized paths.

The model’s inference behavior was influenced by the domination weight learned during training, as edge selection was strongly biased by the learned domination-based relationships between nodes. One key difference between the 50- and 200-epoch models appears to lie in the edge directionality and path structure, which became increasingly refined with extended training. Validation using virtual input data demonstrated that the 50-epoch model was sufficient to reconstruct the network, likely due to the simplicity of the input, which included only two goal nodes (Fig. 3). In contrast, inference using real biological input data showed improved performance with the 200-epoch model, presumably because the input was more complex, containing unchanged or noisy parameters, thereby requiring deeper learning to refine edge relevance and directionality (Fig. 4). The 200-epoch model successfully identified intermediate factors, such as AKT, creatine kinase, FOXO3A, mitochondrial morphology, OPA1, and UQCRC2, as common nodes among individuals from the input of 15 gene expression results (Fig. 5). To further evaluate the model performance, we also compared the results of inference using this model with those obtained from DAVID functional annotation analysis^20^. With same 15 input genes, only terms such as ATP-binding and positive regulation of transcription by RNA polymerase II were identified, both with low enrichment scores (1.38 and 1.35, respectively) in GO terms and the UniProt Knowledgebase (Supplementary data.xlsx online). No results were retrieved from KEGG pathways. Because enrichment tools are designed to identify related cellular systems from sets of differentially expressed factors, they cannot capture interindividual variation, identify molecular factors beyond the input genes, or trace paths originating from physiological stimuli. These observations collectively suggest that the present GNN functions as a scientifically interpretable, small-scale inference model tailored to skeletal muscle biology, with sufficient capacity to support individualized network inference across a tissue-specific corpus of published studies.

PageRank centrality indicated that OPA1 was the most relevant factor associated with the mechanisms underlying interindividual variation in responses to exercise, despite being directly connected to only two types of nodes across the common and individual-specific networks. In contrast, UQCRC2 was connected to 36 types of nodes, although its centrality was lower (0.016–0.023 vs. 0.058–0.085 for OPA1). This apparently paradoxical result suggests that OPA1 is linked to a node functioning as a highly connected hub. Indeed, in the common network, OPA1 is connected to MFN2, one of the primary nodes, which itself links to multiple nodes across all individuals and exhibits high centrality (0.047 in Exer #1, 0.039 in Exer #2, and 0.037 in Exer #3). This local loop likely contributed to the elevated centralities of both OPA1 and MFN2. Taken together, these findings suggest that UQCRC2 represents a key intermediate factor potentially explaining interindividual variation in transcriptional responses to acute exercise.

UQCRC2 (ubiquinol-cytochrome c reductase core protein 2) is a component of mitochondrial respiratory chain complex III, essential for the formation of mitochondrial supercomplexes involving complexes I, III, and IV^21,22^. Missense mutations in UQCRC2 have been linked to impaired mitochondrial respiration and inherited human disorders^23–25^. Notably, a previous study reported that high volume of high-intensity interval training significantly increased UQCRC2 protein expression in skeletal muscle, contributing to enhanced ATP production through more efficient formation of respiratory complexes^26^. In the individualized networks inferred by the model, UQCRC2 exhibited variable interactions with distinct downstream nodes across mice, suggesting that its local network context contributed differently to each individual’s gene expression response (Fig. 7). Importantly, the model’s use of domination-based weighting does not imply that UQCRC2 lies upstream in a canonical signaling pathway. Rather, the model hypothesizes directional relationships in which UQCRC2 functions as a dominant influence relative to its connected nodes within the inferred context. For instance, CKMT2, ACTN3, and MYOZ1 were uniquely associated with UQCRC2 in the Exer #3 (Fig. 7), implying that these factors might be directly or indirectly modulated by UQCRC2, even though the precise molecular mechanisms linking them remain unknown. This interpretive framework reflects the core capability of the present GNN model: to propose biologically plausible, yet hypothetical, directional associations based on learned patterns across multiple studies. While the inferred relationships should be interpreted as hypothesis-generating rather than confirmatory, the model successfully produced skeletal muscle-specific network structures that align with experimental outcomes and capture individual-level diversity. Taken together, these findings support the utility of this GNN as a domain-focused inference tool with strong contextual validity for skeletal muscle biology.

### Limitations and future directions

This study introduces a GNN model designed to infer bioreaction-variation networks within a skeletal muscle–specific corpus. The skeletal muscle model represents one example within the broader framework of bioreaction–variation networks. Models for other organs can be constructed using similar procedures. In fact, separating networks by organ is preferable, since the same molecule may play different roles in different tissues. We also emphasize the need for tools that can integrate these organ-specific networks into an inter-organ framework. The primary aim of this study was not to propose a universally optimized model, but rather to demonstrate a customizable model architecture that can be implemented and adapted at a laboratory scale to address specific biological questions. The model presented here serves as a proof-of-concept example of such a design. However, the current implementation has several limitations as shown below:

- The model requires input data that represent differential states between experimental and control conditions in order to infer individualized networks. Consequently, datasets comprising only baseline or resting conditions, without any comparative group, are not applicable for inference with this model. Therefore, the extraction of individual-specific features through inference reflects relative interindividual variation within the cohort, including the control subjects used for comparison.
- Experimental models or analytical parameters for which relevant edges are scarce in the training dataset may lack sufficient connectivity to support robust inference. In such cases, the model may fail to generate appropriate or biologically meaningful network predictions.
- Because numerical embeddings generated by BioBERT do not directly reflect the magnitude of biological changes in a quantitatively interpretable form, it was necessary to incorporate information on the direction of change, specifically by labeling each relation as either an increase or a decrease. This approach allowed the bioreaction-variation described in the contexts to be translated into vector space. However, capturing such directional changes at the individual level cannot rely on statistical significance testing, which is generally not applicable to single-subject data. In the present study, RNA sequencing data were used, and a twofold change threshold, commonly employed in transcriptomic analyses, was applied to assign directional labels. For other data types or analytical contexts, effect size metrics such as Cohen’s *d* may provide a more appropriate basis for estimating individual-level deviations from the comparison group.

## Supplementary Information

Below is the link to the electronic supplementary material.

Supplementary Material 1

Supplementary Material 2

Supplementary Material 3

Supplementary Material 4

## Data Availability

The datasets generated and/or analysed during the current study are available in the GitHub repository, https://github.com/fumikawano-lab/Bioreaction-Variation-Network. The location of each PyTorch graph dataset on private Google Cloud Storage is also provided in the repository.
