# Supplementary material for: A graph neural network model for inferring interindividual variation from experimental biological data
Source: Sci Rep. 2025 Nov 12;15:39680. doi: 10.1038/s41598-025-23320-4 (PMC12612280; doi:10.1038/s41598-025-23320-4)
Supplement: Supplementary file 1 — Supplementary Material 1 [file 41598_2025_23320_MOESM1_ESM.docx]

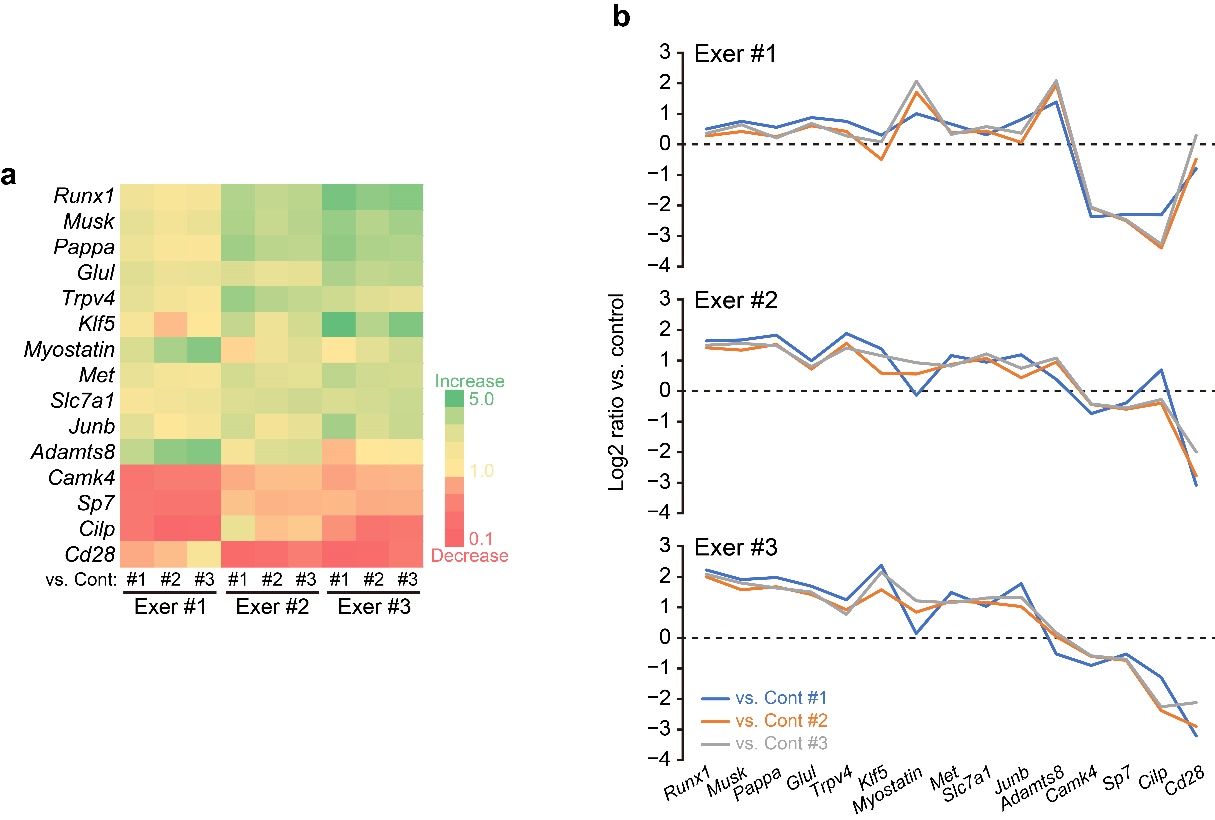


**Figure S1:** Variation in gene responses to a single bout of running exercise in the three exercised mice. (a) Heat map showing the fold changes of 15 genes that exhibited interindividual variation in response to exercise. All possible pairwise comparisons were performed between individuals in the exercised (Exer) and non-exercised (Cont) groups. Genes were ranked from top to bottom according to their average upregulation across the three exercised mice. (b) Line graphs illustrating the response patterns of the same 15 genes in each exercised mouse, presented as log₂ fold changes relative to each control mouse. Note that the three exercised individuals showed distinct patterns of gene expression, highlighting interindividual variation in exercise responses.


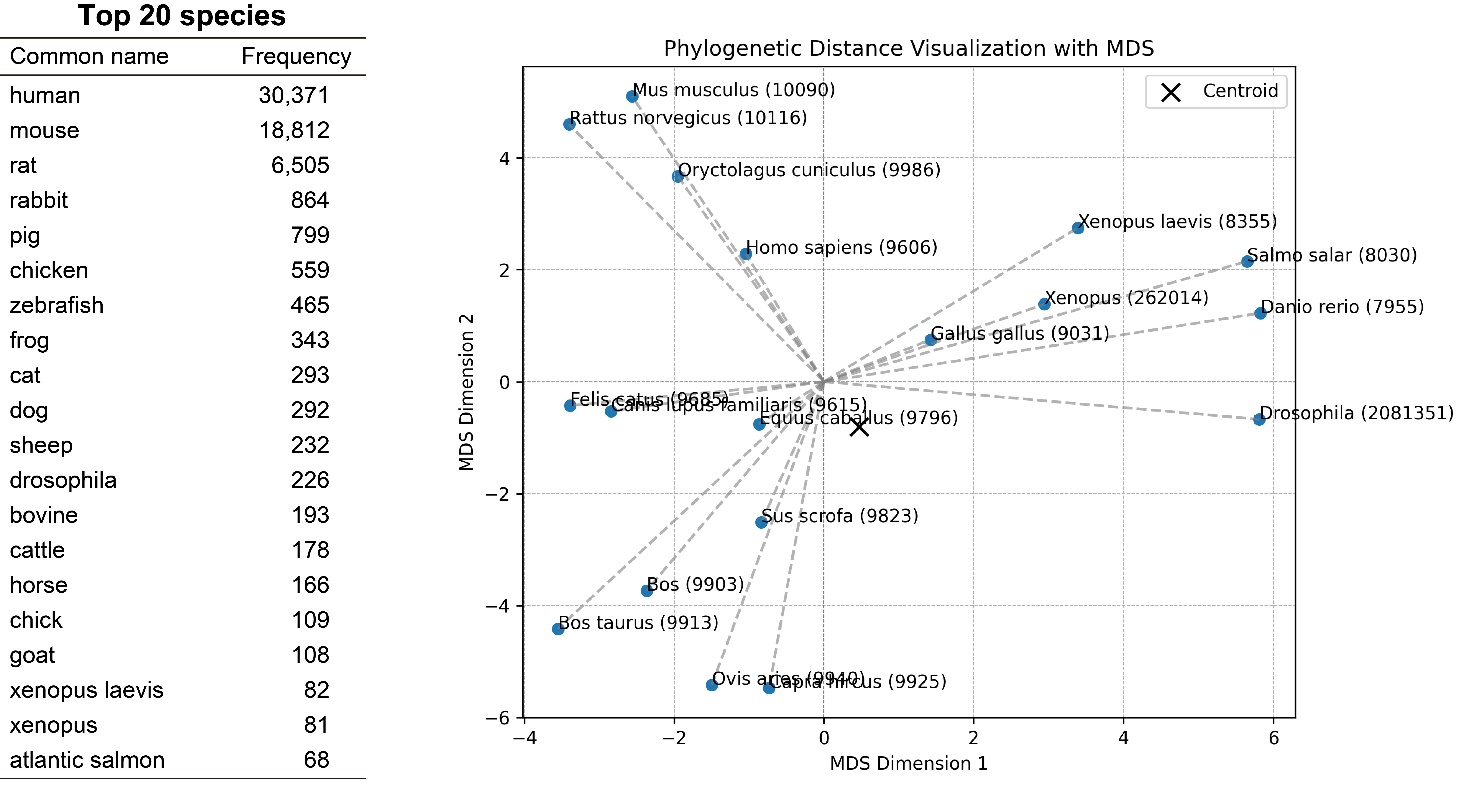


**Figure S2:** Top 20 species most frequently represented in the graph neural network training dataset. Left: Table listing the 20 most common species and their frequencies. Right: Two-dimensional visualization of phylogenetic distances among these species using multidimensional scaling (MDS), based on data from the NCBI Taxonomy database. The centroid (marked with “X”) represents the average phylogenetic position of all species except for human, mouse, and rat.


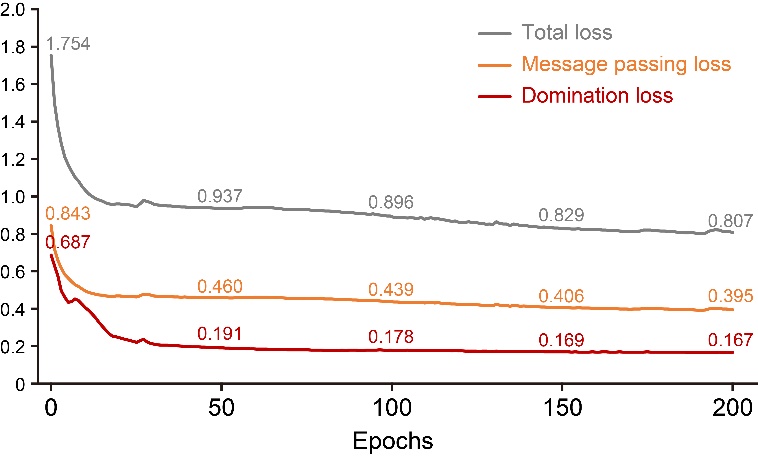


**Figure S3:** Training loss progression over 200 epochs. Line graph showing changes in total loss (grey), message passing loss (orange), and domination loss (red) during training. The total loss was calculated as a weighted sum of the two components: (message passing loss × 2.0) + (domination loss × 0.1).
