# Supplementary material for: A graph neural network model for inferring interindividual variation from experimental biological data
Source: Sci Rep. 2025 Nov 12;15:39680. doi: 10.1038/s41598-025-23320-4 (PMC12612280; doi:10.1038/s41598-025-23320-4)
Supplement: Supplementary file 4 — Supplementary Material 4 [file 41598_2025_23320_MOESM4_ESM.docx]

*Embedding*

The experimental context encoded into vector representations included information related to the experimental models (e.g., species, age, sex, biosample main, biosample detail, experimental type, model main, model detail, and timepoint) as well as target observations (e.g., target, molecular type, analysis main, analysis detail, relation, change, significance, and control). Each set of contextual features was embedded into a 768-dimensional vector representation.

To embed model features, particularly those describing experimental species, species-specific biological variation was incorporated through an angular transformation of the original BioBERT embeddings. Specifically, embeddings corresponding to studies involving human, mouse, and rat were adjusted using Rodrigues' rotation formula. This transformation applied a species-specific angular rotation to the original embedding vector, facilitating species-aligned comparisons during inference. Rotation angles were determined based on phylogenetic distances derived from the NCBI Taxonomy database (see Supplementary Fig. S2 online). Specifically, the positions of the 17 species, which together with human, mouse, and rat comprised the top 20 most frequently represented species in the training dataset, were embedded in a two-dimensional space using multidimensional scaling (MDS), and their centroid was treated as the origin. The angular deviation was defined as the angle between the vector from the origin to each of the three major species (human, mouse, and rat) and a reference axis extending from the origin toward the centroid of the 17 species. The radial distance represented the Euclidean distance from the origin to each species point. These values were then used to normalize the original BioBERT embeddings.

For target features, embeddings were similarly rotated based on the directionality of reported changes. In cases where the "relation" attribute was labeled as “increase” or “decrease,” the embedding vector was rotated +90° or −90°, respectively, using Rodrigues’ rotation formula. This encoding captured the directional semantics of the observed biological changes.

During the graph construction, directed edges were established from the model node to each associated target node within each study. Additionally, undirected edges were formed between all target nodes that were co-analyzed within the same study, resulting in a fully connected subgraph among targets. Each edge was encoded as a 1,536-dimensional tensor by concatenating the 768-dimensional feature embeddings of the source and target nodes. Full implementation details, including preprocessing scripts and embedding procedures, are available via the GitHub repository (https://github.com/fumikawano-lab/Bioreaction-Variation-Network).
